# Perceived identity threat and organizational cynicism in the recursive relationship between psychological contract breach and counterproductive work behavior

**DOI:** 10.1177/0143831X211070326

**Published:** 2022-01-26

**Authors:** Yannick Griep, Samantha D Hansen, Johannes M Kraak

**Affiliations:** Behavioral Science Institute, Radboud University, The Netherlands; Division of Epidemiology, Stress Research Institute, Stockholm University, Sweden; Department of Management, University of Toronto Scarborough and Rotman School of Management, Canada; Management Department, Kedge Business School, France

**Keywords:** Attribution, counterproductive work behavior, identity threat, psychological contract, recursive relationship, violation feelings

## Abstract

Counterproductive work behavior toward the organization (CWB-O) or supervisor (CWB-S) is commonly treated as a consequence of psychological contract breach (PCB). However, drawing from Self-Consistency Theory, the authors in this article argue that the PCB–CWB relationship is recursive through two mediating mechanisms: self-identity threat and organizational cynicism. Furthermore, the authors predict that the relationship between feelings of violation and CWB-O (or CWB-S) would depend on the extent to which the victim attributed blame to the organization (or supervisor). Using weekly and daily survey data, the study found that identity threat was a stronger mediator for recursive CWB–PCB relationships. Moreover, it was found that PCB related positively to violation feelings, which in turn related positively to CWB-O and CWB-S over time. As predicted, the former was moderated by organizational blame attributions, whereas the latter was moderated by supervisor blame attributions. The authors discuss the theoretical implications and propose novel practical implications based on these reciprocal findings.

## Introduction

Upon organizational entry, individuals form a psychological contract (PC) with their organization when they establish beliefs about the reciprocal obligations between themselves and their employer (Rousseau, 2001). The PC is considered a critical construct in the organizational behavior literature because employees who perceive that their employer does not meet its obligations – termed PC breach (PCB) – may develop a negative affective reaction – termed violation feelings (Morrison and Robinson, 1997). Although substantial empirical progress (for a meta-analysis see Zhao et al., 2007) has been made in understanding the relationship between PCB, violation feelings and employee reactions, PC research has remained predominantly contemporaneous and has overlooked the temporal context in which PCB and employee reactions are interrelated and potentially reinforcing each other over time (for a general critique see Hansen and Griep, 2016).

This contemporaneous way of studying PCB, violation feelings and employee reactions is problematic because in doing so scholars have missed an important theoretical tenet of PC Theory (Hansen and Griep, 2016; Rousseau et al., 2018; Tomprou et al., 2015) and Social Exchange Theory (Blau, 1964), namely the fact that perceptions of PCB are situated in time and are perceived with reference to past actions and future anticipations about the mutual exchange relationship (Kozlowski, 2009; Tomprou et al., 2015). As a consequence of this theoretical oversight, scholars have generated the widely held assumption that employee reactions hold the same relationship with PCB at any given point in time; CWB forms the endpoint of employee reactions to PCB, similar to a ‘catharsis’. Not only is such a static ‘antecedent-consequence’ way of thinking in violation with the dynamic conceptualization of the PC (see Hansen and Griep, 2016), it also overlooks the notion that past interactions are an important contextual factor steering employee perceptions of future PCBs.

In the current article, we therefore have the objective of studying the potential for a recursive relationship between PCB and one important behavioral consequence: counterproductive work behavior (CWB). CWB is defined as volitional employee behavior that violates significant organizational norms and is contrary to the organization’s legitimate interests, threatens the well-being of the organization (CWB-O), its individual members (CWB-I), or supervisors (CWB-S) (Fox and Spector, 1999; Jones, 2009).^1^ We focus on CWB because this type of retaliatory behavior is typically associated with a high economical (i.e., estimated annual cost can be as high as 200 billion dollars; Govoni, 1992) and human capital cost (i.e., nearly 69% of supervisors have been a victim of CWB-S; Geddes and Baron, 1997). However, if the PCB–CWB relationship is indeed reciprocal in nature, these estimates may just be scratching the surface of the true costs associated with CWB. Hence, we extend the unilateral vision on the PCB–CWB relationship by drawing on Self-Consistency Theory (Korman, 1970) to suggest that employees do not only engage in CWB in response to PCB, but that employees who have engaged in CWB-O/S will also seek feedback to reconfirm their negative self-conceptions, and by doing so will be more likely to perceive PCB. By recognizing that employees’ current reactions might equally well serve as antecedents to future PCB perceptions, we underscore the interrelatedness of PCB and CWB. Therefore, our first associated research question is: what mechanisms do employees use to reconfirm their negative self-conceptions and how do they explain the reciprocal PCB–CWB relationship?

In addition, we also recognize that there has been a growing trend within the organizational justice, social exchange and CWB literatures to acknowledge that employees maintain distinct perceptions about, and direct different attitudes and behaviors toward, multiple foci in the exchange relationship (for a review see Lavelle et al., 2007). We thus recognize that the target of CWB depends on who the employee determines to be at fault for the PCB and its ensuing violation feelings and therefore liable for action. Hence, we explore the role of blame attributions to understand the circumstances under which employees decide to retaliate toward their organization (CWB-O) or supervisor (CWB-S). In doing so, we are able to better understand employee behavior in the aftermath of PCB and may provide opportunities for more nuanced interventions. We therefore framed our second associated research question as: what is the role of blame attributions in understanding employees’ desire to retaliate toward either their organization or their supervisor when perceiving PCB?

In what follows, we review the relevant literature and develop our hypotheses for this two-study article. In Study 1, we collected five weekly surveys among Belgian volunteers to (1) investigate the mediating role of violation feelings in the PCB–CWB-O relationship, and (2) the direct reverse relationship between CWB-O and PCB. In Study 2, we collected 10 daily surveys among paid US employees to (1) replicate the reciprocal PCB–CWB-O/S relationship, and (2) investigate the moderating role of organizational and supervisor blame attributions. At this point it is also noteworthy to point out that PC Theory (Hansen and Griep, 2016; Rousseau et al., 2018; Tomprou et al., 2015) and Self-Consistency Theory (Korman, 1970) explicitly focus on within-person processes. For example, in their dynamic phase model of PCs, Rousseau and colleagues (2018) argue that the PC and reactions toward disruptions in the PC are idiosyncratic, meaning that when an employee experiences a PCB, he or she is expected to reciprocate this negative event with negative attitudes and/or behaviors. Similarly, a central tenet of Self-Consistency Theory (Korman, 1970) is an individual’s desire for a positive self-view and a relentless drive to defend him/herself against acts that threaten this positive self-view. In other words, these examples highlight that the underlying theory posits what will happen *within* a given individual (i.e., intra-individual processes), but not across a *set* of individuals (i.e., inter-individual processes). Although both PC Theory and Self-Consistency Theory posit within-person processes, the vast majority of research conducted to empirically evaluate these theories often involves the collection and analysis of between-person data, which is not suited to evaluate the theoretically proposed within-person processes (Molenaar, 2004). Fortunately, there is a growing recognition among PC scholars that greater emphasis must be placed on the study of within-person processes and that this can only be accomplished through the study of intra-individual differences (e.g., Conway and Briner, 2002; Griep and Vantilborgh, 2018a, 2018b; Griep et al., 2016; Solinger et al., 2016). In this two-study paper we will thus rely on the study of within-person processes to fully evaluate the theories underlying our hypotheses.

## Theoretical framework and hypotheses

### PCB, violation feelings and CWB

Traditionally, behavioral changes in reaction to PCB have been understood based on Social Exchange Theory (Blau, 1964) and the norm of reciprocity (Gouldner, 1960). These frameworks stipulate that employees and employers engage in a mutual exchange relationship in which each party reciprocates the other party’s contributions by altering their own contributions either in a positive or negative way. In line with the negative norm of reciprocity, perceiving unfair treatment from the organization (i.e., PCB) will most likely be repaid with unfair treatment on behalf of the employee (i.e., CWB-O/S). This PCB–CWB relationship is theorized (Morrison and Robinson, 1997) to unfold via violation feelings. Zhao and colleagues (2007) have indeed found meta-analytical evidence, based on cross-sectional data, for the mediating role of violation feelings in the positive relationship between PCB and a range of negative attitudinal and behavioral outcomes, such as CWB. In this study we base our analysis on these theoretical arguments and a wealth of existing, mainly cross-sectional, empirical evidence (see among others Bordia et al., 2008, 2010; Griep et al., 2020; Restubog et al., 2007, 2015; Suazo and Stone-Romero, 2011) in an aim to extend this work by hypothesizing that violation feelings will also mediate the positive relationship between PCB and CWB-O/S when accounting for the unfolding nature of these relationships over time.

*Hypothesis 1:* At the within-person level, violation feelings (Time X) mediate the positive relationship between (a) PCB (Time X-1) and CWB-O (Time X+1) and between (b) PCB (Time X-1) and CWB-S (Time X+1).

### Multi-foci equals multi-retaliation: The role of blame attributions

We focus on blame attributions to understand how employees maintain distinct perceptions about, and direct different retaliation behaviors toward, multiple foci such as their organization and supervisors (for a review see Lavelle et al., 2007). Blame attributions have been defined as an evaluative judgment in which fault and liability for action are directed toward the entity deemed responsible for the negative consequences of that action (Shaver, 1985). Once employees recognize who is responsible for the experienced violation feelings (i.e., organization or supervisor), they decide who to take action against by engaging in either CWB-O or CWB-S, respectively. Despite increasing recognition of the fact that employee reactions may differ depending on the target, only a handful of studies has examined attributions in the PC. For example, Chao and colleagues (2011) found that employees who attributed a PCB to causes beyond the organization’s control reported lower levels of CWB. Turnley and colleagues (2003) and Ahmed and colleagues (2020) found that employees who perceived a PCB were more likely to reduce their citizenship behaviors when they believed that the organization was to blame for the PCB. Similarly, Costa and Neves (2017) found that employees who blame their organization for a PCB were more likely to reduce their citizenship behaviors through decreased affective organizational commitment, whereas such a reduction was not observed when they blamed the economic context for the PCB. Moreover, Lester and colleagues (2001) found that employees, in general, are more likely to blame their organization in the face of a PCB. Finally, Du and Vantilborgh (2021) found that supervisors were seen as directly responsible, and thus to blame, for most of the employees’ psychological contract evaluations.

Despite the novelty and interesting nature of these findings, it is important to note that various entities (e.g., the organization, the supervisor; Marks, 2001) can cause a PCB, and thus can be the target of action (Lavelle et al., 2007). According to a review of multiple agents in Social Exchange Theory by Lavelle and colleagues (2007), employees are expected to direct their corrective action to the entity that is to blame; one will engage in CWB-O when blame is attributed to the organization, whereas one will engage in CWB-S when blame is attributed to the supervisor. We only found one longitudinal study (Conway et al., 2014) that contrasted such a target similarity (i.e., behavioral reactions are targeted to the entity deemed responsible for an event) versus a spillover (i.e., behavioral reactions are targeted to other entities, who were initially not deemed responsible for an event) approach. This study found that increases in organizational change predicted PCB, which in turn predicted decreases in employee contributions towards the organization, whereas employee contributions towards co-workers and public service users remained unaffected; in line with a target similarity approach to the PCB–CWB relationship. Moreover, Conway and Briner (2002) argued that the perceived responsibility of the organization to deliver upon its obligations may result in the development of organizational blame attributions, which may cause employees to funnel their negative emotions into negative attitudes or behaviors targeting the organization. Using the same rationale, we argue that the development of supervisor blame attributions may result in negative attitudes or behaviors targeting the supervisor. Therefore, we propose that if an employee attributes blame towards the organization, that employee is expected to engage in CWB-O, whereas attributing blame to the supervisor is expected to result in the enactment of CWB-S.

*Hypothesis 2:* At the within-person level, organizational blame attributions (Time X) moderate the positive relationship between violation feelings (Time X) and CWB-O (Time X+1), such that violation feelings lead to CWB-O when attributed to the organization.
*Hypothesis 3:* At the within-person level, supervisor blame attributions (Time X) moderate the positive relationship between violation feelings (Time X) and CWB-S (Time X+1), such that violation feelings lead to CWB-S when attributed to the supervisor.

### Recursive relationships between CWB and PCB: Two mediating mechanisms

Although conceptualized as a dynamic construct, most existing PC research has maintained a very static view of the PC, and in doing so has failed to account for the temporal context which defines the way employees perceive PCB and adjust their acts of CWB accordingly. Moreover, perceptions of PCB do not happen in a vacuum, but are situated in time and happen with reference to past employee or employer actions and future anticipations (Kozlowski, 2009; Tomprou et al., 2015). Over time, a growing number of scholars have drawn attention to this dynamic nature of the PC (e.g., Griep and Vantilborgh, 2018a, 2018b; Griep et al., 2019; Hansen and Griep, 2016; Schalk and Roe, 2007; Solinger et al., 2016; Tomprou et al., 2015; Van der Schaft et al., 2020) leading us to consider what the PCB–CWB relationship might look like if explored as a cycle of processes over time.

To theorize this recursive PCB–CWB relationship, we rely on Self-Consistency Theory (Korman, 1970) and focus on how an employee’s self-consistency cognition will influence perceptions of PCB. The core assumption of Self-Consistency Theory (Korman, 1970) pertains to the fact that individuals are invested in preserving their firmly held positive self-conceptions through soliciting self-consistent feedback. Central to this process is the desire for a positive self-view and a relentless drive to defend themselves against acts that threaten this positive self-view. This idea of self-consistency is also a central theme in the updated dynamic phase model of psychological contract processes (Rousseau et al., 2018) in which the authors argue that particularly goal consistency is crucial to maintaining a well-functioning PC. We however argue that engaging in CWB-O/S endangers one’s positive self-view because acts of CWB, by nature, violate norms and harm others (Fox and Spector, 1999). Hence, despite already having blamed the organization or supervisor for the violation feelings, a new conflict arises between an employee’s desire to have a positive self-view and that employee’s enactment of CWB. We argue that this conflict can be resolved in two ways: (1) perceiving self-identity threat or (2) becoming more cynical towards the organization.

#### The role of perceived identity threat

Enactment of CWB-O/S conflicts with an employee’s desire to have a positive self-view, which potentially threatens that employee’s identity. Indeed, Andersson and Pearson (1999) argue that enactment of behavior with the intention to harm another party is rarely a spontaneous act but more often the culmination of escalating patterns of negative interaction between individuals. That is, they argue that the negative actions of one party (i.e., acts of CWB) lead to negative actions by the other party,a or at least the perception thereof (i.e., perceptions of PCB). Building on this, we argue that acts of CWB do not only happen in response to PCB but may also serve as a precursor to perceptions of PCBs at a later point in time, via perceived identity threat. This can be further supported by drawing from the work by Inzlicht and Kang (2010). These scholars argued that perceiving threat consumes personal resources. In other words, perceiving identity threat could reduce one’s ability to reflect upon and potentially reappraise a discrepancy in one’s PC as one that still falls within one’s zone of acceptance. With fewer cognitive resources, an employee might be more likely to interpret a discrepancy between an organizational obligation and the actual delivered inducement as a PCB (Schalk and Roe, 2007).

*Hypothesis 4:* At the within-person level, identity threat (Time X) mediates the positive relationship between (a) CWB-O (Time X-1) and PCB (Time X+1) and (b) CWB-S (Time X-1) and PCB (Time X+1).

#### The role of organizational cynicism

We propose that organizational cynicism could be an alternative means to protect the self from the inconsistency between engaging in CWB and the desire to maintain a positive self-view, by externalizing the conflict. That is, an employee who perceived a PCB and experienced violation feelings to the point that it changed his/her behavior (i.e., increased enactment of CWB) is likely to develop cynicism, particularly when such experiences are repeated. In turn, those who develop cynicism will be more likely to perceive PCBs at a later point in time because such situations will be consistent with their pre-existing beliefs (i.e., organizational cynicism). This claim is further supported by several studies that highlighted the self-preserving nature of organizational cynicism. For example, Kanter and Mirvis (1989: 2) stated that: ‘cynics at work deeply doubt the truth of what their management tell them and believe that their companies, given a chance, will take advantage of them’. In addition, Dean and colleagues (1998) argued that organizational cynicism is characterized by negative and disparaging attitudes and behaviors toward the organization. Such attitudes are often characterized by lower levels of trust in the organization, which have been found to relate positively to perceptions of PCB at a later point in time (e.g., Robinson, 1996). Some preliminary findings indeed seem to support the notion that cynicism plays an important role in the relationship between PCB and enactment of CWB (see for example Bari et al., 2020; Griep and Vantilborgh, 2018b).

*Hypothesis 5:* At the within-person level, organizational cynicism (Time X) mediates the positive relationship between (a) CWB-O (Time X-1) and PCB (Time X+1) and (b) CWB-S (Time X-1) and PCB (Time X+1).

## Method Study 1

In Study 1 we aimed to establish initial empirical evidence for the reciprocal PCB–CWB relationship by conducting a weekly survey study among Belgian volunteers. Although there are some differences between volunteers and paid employees in terms of PC contents (e.g., ideological inducements are more central to volunteers’ PC), scholars agree that both react similarly when perceiving a PCB (e.g., Bingham, 2005; Griep et al., 2016; Nichols and Ojala, 2009). Moreover, previous studies (e.g., Griep et al., 2016) found that volunteers engage in CWB when perceiving a PCB; thereby contrasting the often-proposed argument that volunteers are not bound to their organization by the usual ties of employment and simply leave the organization when confronted with PCB.

### Procedure

We conducted this study among volunteers working for a Belgian fair-trade organization. Volunteers working for this organization were mainly responsible for sales (running and operating more than 200 points of sales), campaigning, organizing fund-raising events, and external communication to the wider community. We translated all survey items to Dutch and had three colleagues back-translate the items into English. Inconsistencies between the translation and back-translation were discussed and resolved. We recruited our respondents via email and asked them to complete a single general survey prior to completing five consecutive weekly surveys.^2^ The weekly surveys were sent every Friday at 11 a.m. with a due date of Sunday 11 a.m. of the same week. We coded responses as missing data when participants failed to (timely) complete the survey. These weekly measures were frequent enough to capture meaningful changes, but not so frequent that volunteers would likely drop out. We used five weekly surveys because previous work by Solinger and colleagues (2016) indicated that respondents considered a PCB as relevant and related to their functioning for, on average, 2.13 weeks. However, because volunteers often engage in their volunteer activities at irregular intervals and consequently may have had less ‘opportunities’ to perceive a PCB, we aimed to cast the widest possible net to capture PCBs by using five weekly surveys.

### Participants

We contacted 2403 volunteers, of whom 905 respondents clicked the link to the survey, 610 respondents started the survey, and 386 respondents completed the general online survey (response rate_relative to invited_ = 16.06%; response rate_relative to clicked on link_ = 42.65%; response rate_relative to started survey_ = 63.28%). Further, 192 completed the minimum of two consecutive weekly surveys to test our temporal hypotheses. The unit of analysis is ‘weekly surveys’ rather than ‘respondents’ (Conway and Briner, 2002), resulting in an effective sample size of 736 observations (192 respondents × completed weekly surveys). This sample size exceeds the minimum required Level 2 sample size of 30 respondents (Maas and Hox, 2005) needed to make accurate estimates of standard errors in multilevel research.

Our respondents were, on average, 56.02 years old (*SD* = 12.05), 72.90% were female, 68.70% obtained a higher educational degree, 8.00% had managerial responsibilities in their voluntary work, and the average tenure was 11.84 years (*SD* = 8.52). Volunteers spent on average 2.02 days per week (*SD* = 1.40, median = 1.98 days) or 2.85 hours per day (*SD* = 1.95) on their volunteer activities. Only a small percentage of volunteers spent entire days on their voluntary activities whereas the vast majority spent a few hours per day or week. We found that dropout could only be explained by age; older respondents were less likely to drop out between the general and weekly surveys (β = –.47, SE = .002, *p* < .001) and during the weekly surveys (β = –.37, SE = .001, *p* < .001).

### Measures

#### General survey measures

We used the general online survey to collect demographic information on respondents’ age (in years), gender (female or male), educational background (highest level of formal education), company tenure (in years), and the level of promised inducements. *Level of promised inducements* was assessed to confirm that inducement types were relevant to this particular sample. Respondents rated the extent to which their employer promised to provide them with each of 20 items on a 5-point scale (1 = minimally or not at all, 5 = to a very large extent; for a similar approach see Montes and Irving, 2008). These items represented the most widely studied types of inducements: transactional and relational (see PSYCONES, 2005), as well as ideological inducements (see Bingham, 2005), which are particularly relevant to the volunteer context and relate to reasons why one engages in voluntary activities. The means for the transactional (α = .74), relational (α = .76), and ideological (α = .94) promises ranged from 3.21 to 4.00, 4.23 to 4.67, and 4.35 to 4.80, respectively, indicating that these inducements were all relevant for these volunteers.

#### Weekly survey measures

Consistent with similar diary studies (e.g., Conway and Briner, 2002), we used shortened scales to ensure a reasonable survey length and to limit dropout. Scales were counterbalanced to rule out potential order effects in the results. Further, to reinforce the due date and ensure fit within a volunteer context, all items were worded such that they: (1) included ‘*during the past week*’, (2) used the past tense, and (3) referred specifically to volunteer activities. A full list of items for each measure are given in the Appendix.

*PCB* was measured by presenting the same list of 20 PC items. In line with the recommendations by Conway and Briner (2002) and Griep and colleagues (2016), we asked respondents to reply ‘yes’ or ‘no’ to the question: ‘Did your organization breach one or more of the following obligations during the past week?’ for each of the PC inducements. Similar to previous studies (e.g., Conway and Briner, 2002; Griep et al., 2016; Robinson and Rousseau, 1994) we chose to assess PCB globally as opposed to assessing various facets of PCB to keep the survey length at a reasonable level while also maximizing the probability of capturing PCB (i.e., we likely could not capture many PCBs if we only inquired about two or three PC inducements). Respondents reported a total of 863 specific PC breaches over the course of the study: 113 respondents reported 247 transactional PCBs (*M* = 2.33; *SD* = 1.40), 99 respondents reported 231 relational PCBs (*M* = 1.79; *SD* = 0.95), 99 respondents reported 385 ideological PCBs (*M* = 3.89; *SD* = 2.72) over the course of the study. Previous studies (e.g., Griep et al., 2016) found that this single item correlated significantly with Robinson and Morrison’s (2000) PCB and violation feelings measure, confirming its validity.

*Feelings of violation* were measured with a single item: ‘To what extent did the breach of this (these) obligation(s) have a negative emotional effect on you during the past week?’ Responses ranged from (0) ‘not at all’ to (5) ‘to a very great extent’ (Solinger et al., 2016). All respondents who did not perceive a PCB indicated (0) ‘not at all’; aligning with theoretical arguments that violation feelings are conditional upon PCB (Morrison and Robinson, 1997). Previous studies (e.g., Griep et al., 2016) found that this item correlated significantly with Robinson and Morrison’s (2000) PCB and violation feelings measure, confirming its validity.

*CWB-O* was measured with a 6-item scale (Dalal et al., 2009). Respondents indicated (‘yes’ or ‘no’) whether they purposefully engaged in any of the presented behaviors during the past week. An example item is: ‘worked slower than necessary’. CWB-O scores ranged from 0 to 6. This measure is a formative construct, rendering the estimation of an internal reliability coefficient obsolete (Coltman et al., 2008).

### Analytic strategy

Using a single reciprocal mediation model, we predicted (1) the intensity of violation feelings based on the number of reported PCBs during the previous week, (2) CWB-O during one week, based on the intensity of violation feelings during the previous week, and (3) the number of PCBs during one week, based on CWB-O during the previous week, across all weeks of data collection. We estimated the indirect effect from the number of PCBs on CWB-O via violation feelings using the product of coefficient approach. We scrutinized the significance of these indirect effects by means of the Monte Carlo Method (Bauer et al., 2006) using the INTEGRATION = MONTECARLO (10,000) option in Mplus 7.4, thereby generating 95% confidence intervals (95% CI). We compared a partial versus full mediation model using the 2Log Likelihood (–2LL) difference test. Because our data had a nested structure, we estimated intra-class correlation coefficients (ICC) of PCB, violation feelings, and CWB-O to assess the need for a multilevel model (Hox, 2010). Results indicated that the largest proportion of the variance (ICCs are .30, .30, .17, respectively) could be attributed to within-person differences. Therefore, we relied on the strategy of person mean-centered to investigate the proposed within-person processes and fully evaluate the theoretical frameworks drawn upon.

## Results Study 1

### (Multilevel) confirmatory factor analysis (CFA)

Although not directly pertinent to our hypotheses, we did perform a CFA at the between-person level to ensure relevance of the transactional, relational and ideological items for this sample. We used Dyer and colleagues’ (2005) conventional standards to assess model fit and compared competing models using log likelihood ratio tests. The theorized 3-factor model fit the data well, with each PC item loading significantly, and in the expected direction, onto its respective latent factor (Table 1). Alternative models A (Δχ^2^(2) = 57.09, *p* < .001), B (Δχ^2^(2) = 32.84, *p* < .001), C (Δχ^2^(2) = 107.29, *p* < .001) and D (Δχ^2^(3) = 166.23, *p* < .001) fit the data significantly worse. Hence, we are confident that the transactional, relational and ideological items can be used to assess volunteers’ perceptions of promised inducements. Next, we tested whether PCB, violation feelings and CWB-O can be empirically distinguished from each other using a series of multilevel CFAs. Because our CWB-O measure was dichotomous, we used the WLSMV estimator and reported the Weighted Root Mean Square Residual (WRMR) fit index. We compared a theoretical model containing three first-order latent factors to four alternative models (see Table 1). Although alternative model A fitted equally well to the data as the theoretical model, not all items loaded significantly on their latent factor, and several fit indices did not reach the suggested cut-off values. Moreover, it should be noted that both the AIC and sample-size adjusted BIC values favor the theoretical model (AIC = 4369.37; sample-size adjusted BIC = 4387.81) over the alternative model A (AIC = 4399.85; sample-size adjusted BIC = 4421.96). Alternative model B (Δχ^2^(1) = 25.10, *p* < .001), C (Δχ^2^(1) = 25.10, *p* < .001) and D (Δχ^2^(1) = 25.10, *p* < .001) fit the data significantly worse. Hence, our 3-factor theoretical model (RMSEA = .03, CFI = 1.00, TLI = 1.00, WRMR = .57) guided hypotheses testing.

**Table 1.** Results from (multilevel) confirmatory factor analyses (Study 1).

Model	χ^2^(*df*)	RMSEA	CFI	TLI	WRMR	SRMR_between_
*Confirmatory factor analyses*						
Theoretical model	464.44 (167)	.08	.90	.85	-	.08
Alternative model A	407.35 (169)	.09	.85	.83	-	.09
Alternative model B	497.28 (169)	.10	.79	.76	-	.13
Alternative model C	571.80 (169)	.11	.76	.73	-	.14
Alternative model D	630.67 (170)	.12	.70	.67	-	.15
*Multilevel confirmatory factor analyses*						
Theoretical model	15.10 (19)	.03	1.00	1.00	.57	-
Alternative model A	15.85 (19)	.03	1.00	1.00	.59	-
Alternative model B	40.20 (20)	.04	.67	.54	.73	-
Alternative model C	40.20 (20)	.04	.67	.54	.73	-
Alternative model D	40.24 (20)	.04	.67	.54	.73	-

*CFA theoretical model*: Transactional, relational and ideological inducements each load onto a separate latent factor. *Alternative model A*: Transactional and relational inducements load onto one latent factor; ideological inducements load onto one latent factor. *Alternative model B*: Relational and ideological inducements load onto one latent factor; transactional inducements load onto one latent factor. *Alternative model C*: Transactional and ideological inducements load onto one latent factor; relational inducements load onto one latent factor. *Alternative model D*: Transactional, relational and ideological inducements load onto one single latent factor. *Multilevel CFA theoretical model*: PCB, violation feelings and CWB-O each load onto a separate latent factor. *Alternative model A*: PCB and violation feelings load onto one latent factor; CWB-O loads onto one latent factor. *Alternative model B*: PCB and CWB-O load onto one latent factor; violation feelings load onto one latent factor. *Alternative model C*: PCB loads onto one latent factor; violation feelings and CWB-O load onto one latent factor. *Alternative model D*: PCB, violation feelings and CWB-O load onto one single latent factor.

### Descriptive results

Table 2 provides an overview of the means, standard deviations and within-person correlations for each weekly survey.

**Table 2.** Means, standard deviations and correlations among the focal variables for weekly survey (Study 1).

	*M*	*SD*	1.	2.	3.
1. PC breach	.91 / 1.08 / 1.82 / .76 / .28	3.05 / 3.08 / 4.06 / 2.68 / 1.77	-	-	
2. Violation feelings	1.96 / 1.90 / 2.08 / 1.58 / 1.43	.99 / .94 / 1.05 / .84 / 1.13	.67^***^ / .60^***^ / .65^***^ / .55^***^ / .38^***^	-	
3. CWB-O	.38 / .79 / .58 / .42 / .43	.65 / 1.08 /.87 / .61 / .79	.03 / .12 / .09 / .15 / .03	. 22^**^ / .21^**^ / .18^*^ / .27^**^ / .06	-

*Notes.* * *p* < .05; ** *p*< .01; *** *p*< .001. Means, standard deviations and correlations are presented for each weekly survey for those observations where respondents reported at least one PC breach in that given week. Weekly survey 1 correlations (*N* = 169); weekly survey 2 correlations (*N* = 163); weekly survey 3 correlations (*N* = 138); weekly survey 4 correlations (*N* = 145); weekly survey 5 correlations (*N* = 121). The means for PC breach refer to the number of reported PC breaches and the means for CWB-O refer to the number of reported acts of CWB-O during a given week.

### Preliminary model testing

Before presenting the results, we would like to point out that we modeled *change* in each variable by controlling for the previous level (i.e., previous week) of the same variable (i.e., auto-correlation). Moreover, we estimated a full and a partial mediation model and compared them using the 2Log Likelihood (–2LL) difference test to assess which of these models had a better fit to the data. The two models did not differ significantly from each other (∆−2LL(1) = 1.62, *p* = .20). However, the full mediation model (sample-size adjusted BIC = 5681.18) fits the data better than a partial mediation model (sample-size adjusted BIC = 5699.84). When comparing models, a difference in BIC of 10 corresponds to the odds being 150:1 that the model with the smaller BIC or sample-size adjusted BIC value is the better fitting model and is considered ‘very strong’ evidence in favor of the model with the smaller BIC or sample-size adjusted BIC value (Raftery, 1995).

### Hypothesis testing

**Figure 1.**
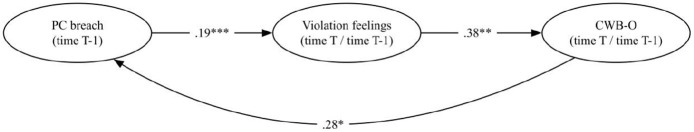
Standardized estimated paths at the within-person level for the proposed full mediation model. *Notes.* * *p* < .05; ** *p* < .01; *** *p* < .001. Results indicate change in each variable by controlling for the auto-correlation at the previous moment in time.

Figure 1 displays the standardized estimated paths at the within-person level for the proposed full mediation model. Results showed that the number of reported PCBs was positively related to the intensity of violation feelings, which in turn was positively related to CWB-O, which in turn was again positively related to the number of PCBs. In addition, we found a significant time-lagged indirect effect of the number of PCBs on CWB-O via the intensity of violation feelings (estimate = .07; 95% CI = [.02; .12]). In sum, these results support Hypothesis 1a and 4a (without mediation).

### Sensitivity analysis

Because some scholars have suggested that individuals may react differently to transactional and relational PCB and fulfillment (e.g., Montes and Irving, 2008), we created a composite score for each PCB dimension (i.e., transactional, relational and ideological) and tested for potential differential effects on the outcomes under study. We compared a model with separate regression parameters for each PCB dimension with a model in which these regression parameters were constrained to be equal and found that the proposed relationships did not differ significantly between the three PCB dimensions (χ^2^(18, *N* = 192) = 5.85, *p* = .99). These findings align with previous arguments (Bordia et al., 2008) and empirical evidence (Griep et al., 2016) that global PCB measures (i.e., either general PCB measures or global composite measures, respectively) are likely better at capturing assessments of PCB in a diary context.

## Discussion Study 1

We found that the number of PCBs is positively related to the intensity of violation feelings, which in turn is positively related to CWB-O. In addition, our results demonstrated that engagement in CWB-O at one point in time also served as an antecedent of PCB at the next point in time. We thus provided initial evidence for a recursive PCB–CWB relationship. Nevertheless, Study 1 is associated with a number of shortcomings which we sought to overcome in Study 2. First, although our measures of PCB and violation feelings have been successfully used in previous studies (e.g., Griep et al., 2016; Solinger et al., 2016) and were found to correlate as expected with traditional measures of PCB and negative affectivity (see Griep et al., 2016), the dichotomous nature of our PCB measure left little room for nuance in respondents’ perceptions of PCB, and our violation feelings measure focused on general negative emotion instead of adopting the complete feelings of violation scale. Hence, Study 2 deployed the widely used global breach and violation feelings scales developed by Robinson and Morrison (2000) to conceptually replicate the proposed relationships and to better capture this mixture of negative emotions. Conceptual replication allows researchers to re-test the same theoretical idea or hypothesis repeatedly, while using different populations, different ways of manipulating variables, different ways of measuring variables, or using different study designs or a combination thereof. In doing so, it allows researchers to demonstrate reliability and validity of a hypothesis while advancing scientific knowledge (see Van Berkel and Crandall, 2018). Second, because it is quite possible that employees may retaliate against a different target depending on who they blame for their feelings of violation, we included measures of CWB-O and CWB-S, as well as measures of organizational and supervisor blame attributions to test this possibility. In addition, because our initial measure of CWB counted the number of CWB acts but did not allow for meaningful variation in the frequency of these CWB acts, we included behavioral frequency measures of CWB-O/S in Study 2. Third, recollection bias may have affected our results due to a weekly time lag. To overcome this issue, Study 2 included daily time lags. Finally, given the unique nature of the sample in Study 1 (volunteers), we aim to replicate and extend our findings using a diverse sample of paid employees from a variety of organizations.

## Method Study 2

In Study 2, we tested whether violation feelings mediated the relationship between the intensity of PCB and the frequency of CWB-O (Hypothesis 1a) and CWB-S (Hypothesis 1b) in a sample of paid US employees. In addition, we examined the moderating role of blame attribution on the relationship between violation feelings and CWB-O and CWB-S (Hypotheses 2 and 3). Next, we juxtaposed the mediating role of self-identity threat and organizational cynicism in the relationship between the frequency of CWB-O and CWB-S, and the intensity of PCB (Hypotheses 4 and 5).

### Procedure

We recruited respondents via StudyResponse, an online paid participant panel. StudyResponse reached out to a random selection of their registered panel members and invited them to take part in this specific study. Participation in this study was voluntary and interested respondents completed a general online survey and were later sent a short daily survey at 4 p.m. for 10 consecutive working days. We used daily surveys because most paid employees, in contrast to volunteers in Study 1, have a full-time contract and thus can potentially perceive a PCB at the daily level. We selected a two-week period for daily surveys based on recommendations by Wheeler and Reis (1991), who contend that a two-week record-keeping period represents a stable and generalizable estimate of social life. We requested our respondents to return the survey by midnight on the day they received the survey invitation. Failure to complete surveys or return them on time was treated as missing data. Respondents received a $2 Amazon gift certificate for each completed daily survey.

Although it could be argued that respondents from an online paid participant panel are primarily motivated by payment and hence do not respond honestly or reliably to the survey questions, we would like to point out that, consistent with empirically-based recommendations (e.g., Huang et al., 2012), we took steps to identify and exclude careless responders: (1) all respondents were told that only truthful and accurate responses would be compensated, (2) we included ‘check items’ (e.g., for this item, please indicate ‘totally agree’) throughout each survey to identify and remove respondents who failed to correctly complete these check items, and (3) we excluded participants whose time stamps did not reflect honest responding (e.g., completed the survey in a few seconds). As a result of these checks, we removed 1 respondent from the general survey and 15 respondents from the daily surveys. Buhrmester and colleagues (2011) demonstrated that the data obtained from Amazon’s Mechanical Turk – a similar online paid participant panel – are at least as reliable, and in some cases even more reliable, as those obtained via traditional sampling methods and are not affected by realistic compensation rates.

### Participants

Of the 142 invited, 114 respondents completed the general survey (response rate = 80.28%) and 102 (response rate = 71.83%) completed the minimum of two consecutive daily surveys (these response rates already account for the removal of careless responders). The effective sample size included 921 observations (102 respondents × completed daily surveys) or an average of 9.03 (*SD* = 1.64) completed daily surveys per respondent. Our respondents were, on average, 49.79 years old (*SD* = 10.81), 40.60% were female, 49.60% obtained a higher educational degree, 89.60% had a permanent full-time job, 35.80% had managerial responsibilities, and the average company tenure was 12.19 years (*SD* = 8.82). On average, our respondents worked for 39.67 hours per week (*SD* = 9.04). Our respondents came from the following sectors: 10.90% manufacturing, 11.50% wholesale and retail trade, 5.90% transportation and utilities, 2.20% information, 7.80% financial activities, 13.20% professional and business services, 23.40% education and health services, 9.90% leisure and hospitality, 5.70% public administration, and 9.50% other services. Logistic regression analyses indicated that none of the demographics nor the variables under study explained dropout during the daily surveys.

### Measures Study 2

#### General survey measures

As in Study 1, the general survey was used to collect demographic information, assess promised inducements, and measure general negative affectivity. *Level of promised inducements* was assessed for similar reasons and in a similar way as in Study 1 (see relational and transactional items). We omitted ideological PC inducements as they are far less relevant to a sample of paid employees. The means for the transactional (α = .82) and relational (α = .91) PC inducements ranged from 2.83 to 3.36, and 3.08 to 3.39, respectively. As expected, the proposed transactional and relational PC inducement model (RMSEA = .09, CFI = .90, TLI = .90, SRMR = .07; Dyer et al., 2005) fitted the data significantly better than an alternative single inducement model (Δχ^2^(1) = 3.88, *p* < .05).

#### Daily survey measures

*Psychological contract breach* was measured using the 5-item PCB scale by Robinson and Morrison (2000). An example item is: ‘During the past day, I have not received everything promised to me in return for my contributions’. Respondents rated the extent to which they agreed with each of the statements on a 7-point Likert scale ranging from (1) ‘totally disagree’ to (7) ‘totally agree’. The level-specific within-person (ω = .75) omega reliability (Geldhof et al., 2014) was satisfactory.

*Violation feelings* were measured using the 4-item scale by Robinson and Morrison (2000). An example item is: ‘During the past day, I felt a great deal of anger toward my organization’.

Respondents rated the extent to which they agreed with each of the statements on a 7-point Likert scale ranging from (1) ‘totally disagree’ to (7) ‘totally agree’. The level-specific within-person (ω = .91) omega reliability was satisfactory.

*Organizational and supervisor blame attributions* were measured by presenting the same 5- item PCB scale by Robinson and Morrison (2000) and asking our respondents to indicate on a 7-point scale, ranging from (1) ‘minimally or not at all’ to (7) ‘to a very great extent’, the extent they blamed their (1) organization and (2) supervisor. The level-specific within-person (ω = .71 and ω = .63, respectively) omega reliability was satisfactory.

*CWB-O and CWB-S* were measured with six items each (Dalal et al., 2009). An example item of CWB-O is: ‘During the past day, I purposefully did not work to the best of my ability’. An example item of CWB-S is: ‘During the past day, I purposefully tried to harm my superior’.

Respondents rated the frequency of these behaviors on a 7-point Likert scale ranging from (1) ‘minimally or not at all’ to (7) ‘to a very great extent’. The level-specific within-person (ω = .86 and ω = .88, respectively) omega reliability was satisfactory.

*Identity threat* was measured with a 4-item identity threat scale (Breakwell, 1986). An example item is: ‘During the past day, I felt that my sense of self-worth was undermined’. Each item was rated on a 6-point Likert scale ranging from (1) ‘very unlikely’ to (6) ‘very likely’. The level-specific within-person (ω = .89) omega reliability was satisfactory.

*Organizational cynicism* was measured with a 12-item organizational cynicism scale (Dean et al., 1998). An example item is: ‘During the past day, when my organization said it was going to do something, I wondered if it would really happen’. Respondents indicated their level of agreement on a 7-point Likert scale ranging from (1) ‘strongly disagree’ to (7) ‘strongly agree’. The level-specific within-person (ω = .91) omega reliability was satisfactory.

A full list of items for each measure for Study 2 is given in the Appendix.

### Analytic strategy

We followed the recommendations of Edwards and Lambert (2007) and simultaneously tested moderation and mediation effects. The moderation effects were tested by including an interaction effect between (1) violation feelings and organizational blame attribution, and (2) violation feelings and supervisor blame attribution, across all days of data collection. To interpret these multilevel moderation relationships, we used the regions of significance approach or the Johnson–Neyman technique (Preacher et al., 2006) instead of the traditional simple slopes method. Despite its broad usefulness, the simple slopes method has an important limitation that largely hampers our ability to interpret the full extent of the interaction: the choices of the conditional values are ultimately arbitrary (i.e., –1SD, mean, +1SD). The Johnson–Neyman technique identifies the full range of the moderator for which the interaction is significant (i.e., all values where the 95% confidence bands do not include zero). While the upper dashed line in such plots indicates the 2.5% upper region boundaries of significance, the lower dashed line indicates the 2.5% lower region boundaries of significance. The solid line in between the confidence bands represents the size and the direction of the relationship between the independent and the dependent variable for different values of the moderator.

The mediation effects were tested by means of the product of coefficients approach and their significance was scrutinized by means of 95% Monte Carlo Confidence Intervals (95% CI; Preacher and Selig, 2012). We linked the regression coefficients of the type of blame attributions, the intensity of violation feelings, and their interaction terms (type of blame attributions × violation feelings) to CWB-O and CWB-S, across all days of data collection. Finally, we tested time-lagged mediation effects by linking CWB-O and CWB-S to PCB via identity threat and organizational cynicism across all days of data collection. We simultaneously tested the pathway from PCB to CWB via violation feelings, as well as the recursive pathway from CWB to PCB via identity threat and organizational cynicism.

We estimated ICCs of PCB, violation feelings, organizational and supervisor blame attribution, CWB-O, CWB-S, identity threat and organizational cynicism because the data had a nested structure (i.e., daily surveys nested within individuals). The largest proportion of the variance in these variables (ICCs are .23, .26, .25, .17, .42, .35, .30, .25, respectively) could be attributed to within-person differences (Hox, 2010). Hence, as in Study 1, we relied on a person mean-centered strategy to investigate the proposed within-person processes and fully evaluate the theoretical frameworks drawn upon. We conducted all analysis in Mplus version 7.1.

## Results Study 2

### Multilevel CFA

We started by performing a series of multilevel CFAs in which we specified the same factor structure at the within-person (i.e., as per our focus on within-person processes) and between-person (i.e., as per the traditional focus of the literature) level. As in Study 1, we used Dyer and colleagues’ (2005) conventional standards to assess model fit and compared competing models using loglikelihood ratio tests. Based on our theoretical expectations and the sensitivity analysis of Study 1 (i.e., indicating a single PCB factor) we compared a theory-based model containing eight first-order latent factors to 12 alternative models (see Table 3). These alternative models were identified based on theoretical (e.g., anthropomorphism of the organization) and empirical (e.g., high correlations at the within- and between-person level) arguments for potential overlap between constructs. Alternative models A (Δχ^2^(8) = 49.31, *p* < .001), B (Δχ^2^(7) = 1865.93, *p* < .001), C (Δχ^2^(7) = 2304.11, *p* < .001), D (Δχ^2^(7) = 1342.20, *p* < .001), E (Δχ^2^(7) = 2171.71, *p* < .001), F (Δχ^2^(7) = 1169.53, *p* < .001), G (Δχ^2^(7) = 165.95, *p* < .001), H (Δχ^2^(7) = 1060.76, *p* < .001), I (Δχ^2^(7) = 1998.89, *p* < .001), J (Δχ^2^(7) = 1054.94, *p* < .001), K (Δχ^2^(7) = 2436.55, *p* < .001), and L (Δχ^2^(7) = 2220.48, *p* < .001) fitted significantly worse to the data than the theory-based model. Hence, our 8-factor theory-based model (RMSEA = .05, CFI = .93, TLI = .92, SRMR_within_ = .06 and SRMR_between_ = .06) guided hypotheses testing.

**Table 3.** Results from multilevel confirmatory factor analyses (Study 2).

Model	χ^2^ (*df*)	RMSEA	CFI	TLI	SRMR_within_	SRMR_between_
Theoretical model	8900.89 (2821)	.05	.93	.92	.06	.06
Alternative model A	8950.20 (2813)	.05	.93	.92	.06	.06
Alternative model B	10766.82 (2828)	.06	.78	.77	.07	.08
Alternative model C	11205.00 (2828)	.06	.76	.76	.07	.08
Alternative model D	10243.09 (2828)	.05	.79	.78	.06	.07
Alternative model E	11072.60 (2828)	.06	.77	.76	.08	.10
Alternative model F	10070.42 (2828)	.05	.80	.79	.07	.08
Alternative model G	9066.84 (2828)	.05	.82	.82	.06	.06
Alternative model H	9961.65 (2828)	.05	.80	.79	.06	.06
Alternative model I	10899.78 (2828)	.06	.77	.77	.09	.12
Alternative model J	9955.83 (2828)	.05	.80	.79	.06	.06
Alternative model K	11337.44 (2828)	.06	.76	.75	.09	.11
Alternative model L	11121.37 (2828)	.06	.77	.76	.08	.09

*Notes. N* (within) = 921, *N* (between) = 102.

*Theoretical model*: PCB, violation feelings, organizational blame attributions, supervisor blame attributions, CWB-O, CWB-S, organizational cynicism and identity threat each load onto a separate latent factor; *Alternative model A*: Transactional PCB, relational PCB, violation feelings, organizational blame attributions, supervisor blame attributions, CWB-O, CWB-S, organizational cynicism and identity threat each load onto a separate latent factor; *Alternative model B*: Violation feelings and CWB-O load onto one latent factor; PCB, organizational blame attributions, supervisor blame attributions, CWB-S, organizational cynicism and identity threat each load onto a separate latent factor; *Alternative model C*: Violation feelings and CWB-S load onto one latent factor; PCB, organizational blame attributions, supervisor blame attributions, CWB-O, organizational cynicism and identity threat each load onto a separate latent factor; *Alternative model D*: Violation feelings and identity threat load onto one latent factor; PCB, organizational blame attributions, supervisor blame attributions, CWB-O, CWB-S and organizational cynicism each load onto a separate latent factor; *Alternative model E*: Violation feelings and organizational cynicism load onto one latent factor; PCB, organizational blame attributions, supervisor blame attributions, CWB-O, CWB-S and identity threat each load onto a separate latent factor; *Alternative model F*: Organizational and supervisor blame attributions, supervisor blame attributions load onto one latent factor; PCB, violation feelings CWB-O, CWB-S, organizational cynicism and identity threat each load onto a separate latent factor; *Alternative model G*: CWB-O and CWB-S load onto one latent factor; PCB, violation feelings, organizational blame attributions, supervisor blame attributions, organizational cynicism and identity threat each load onto a separate latent factor; *Alternative model H*: CWB-O and identity threat load onto one latent factor; PCB, violation feelings, organizational blame attributions, supervisor blame attributions, CWB-S and organizational cynicism each load onto a separate latent factor; *Alternative model I*: CWB-O and organizational cynicism load onto one latent factor; PCB, violation feelings, organizational blame attributions, supervisor blame attributions, CWB-S and identity threat each load onto a separate latent factor; *Alternative model J*: CWB-S and identity threat load onto one latent factor; PCB, violation feelings, organizational blame attributions, supervisor blame attributions, CWB-O and organizational cynicism each load onto a separate latent factor; *Alternative model K*: CWB-S and organizational cynicism load onto one latent factor; PCB, violation feelings, organizational blame attributions, supervisor blame attributions, CWB-O and identity threat each load onto a separate latent factor; *Alternative model L*: Organizational cynicism and identity threat load onto one latent factor; PCB, violation feelings, organizational blame attributions, supervisor blame attributions, CWB-O and CWB-S each load onto a separate latent factor.

### Descriptive results

Table 4 provides an overview of the means, standard deviations, zero-order (i.e., between- person) and person-centered (i.e., within-person) correlations. We performed additional analyses because some of the correlations at the between-person level (despite not being of interest to our hypotheses) appeared high. The results indicated that controlling for unreliability did not substantially increase the correlations. Furthermore, the aforementioned CFA analyses revealed that even the highly correlated scales measured distinct constructs at the between- and within-person level. Finally, it should be noted that due to our approach (i.e., focus on within-person processes and relying on the strategy of person mean-centering), the interpretation of our within-person findings would not be compromised.

**Table 4.** Means, standard deviations, zero-order and person-centered correlations among the focal variables (Study 2).

	*M*	*SD*	1.	2.	3.	4.	5.	6.	7.	8.
1. Psychological contract breach	2.99	.52	-	.22^***^	.09^**^	.09^**^	.06^*^	.10^**^	.02	.01
2. Violation feelings	1.78	1.06	.47^***^	-	.02	.01	.32^***^	.30^***^	.19^***^	.33^***^
3. Organizational blame attribution	4.58	1.18	.47^***^	–.18^*^	-	.41^***^	.06^*^	.08^*^	.03	.01
4. Supervisor blame attribution	4.83	1.31	.47^***^	–.35^***^	.77^***^	-	–.03	–.02	–.02	–.07^*^
5. Frequency of CWB-O	1.76	1.04	.17	.71^***^	–.25^**^	–.04	-	.83^***^	.22^***^	.49^***^
6. Frequency of CWB-S	1.52	.94	.18	.72^***^	–.14	–.39^***^	.79^***^	-	.20^***^	.49^***^
7. Organizational cynicism	2.65	1.39	.51^***^	.77^***^	–.21^*^	–.34^***^	.65^***^	.64^***^	-	.18^***^
8. Identity threat	1.72	1.20	.28^**^	.84^***^	–.11	–.33^***^	.84^***^	.79^***^	.67^***^	-

*Notes.* * *p* < .05; ** *p* < .01; *** *p* < .001. Zero-order (between-person; *N* = 102) correlations are presented below the diagonal, whereas person-centered (within-person; *N* = 921) correlations are presented above the diagonal.

### Preliminary model testing

As in Study 1, we modeled *change* in each variable. Furthermore, as in Study 1, we estimated a full mediation and a partial mediation model and compared them using the 2Log Likelihood (–2LL) difference test to assess which of these models had a better fit to the data. Although the 2Log Likelihood difference test indicated no significant difference between the full and partial mediation model (∆−2LL(2) = 1.76, *p* = .42), the sample-size adjusted BIC value indicated that the full mediation model (sample-size adjusted BIC = 10642.89, RMSEA = .07, CFI = .99, TLI = .94, SRMR = .02) fitted the data better than the partial mediation model (sample-size adjusted BIC = 10672.83, RMSEA = .08, CFI = .98, TLI = .92, SRMR = .04).

### Hypothesis testing

**Figure 2.**
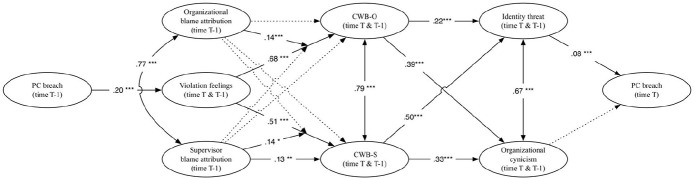
Standardized estimated paths at the within-person level for the proposed moderated mediation model. *Notes.* * *p* < .05; ** *p* < .01; *** *p* < .001. Dotted lines indicate non-significant relationships. Double arrowed lines indicate correlations. Results indicate change in each variable by controlling for the auto-correlation at the previous moment in time.

Figure 2 depicts the results of the within-person level for the proposed moderated mediation model. Our results indicated that the intensity of PCB was positively related to violation feelings. Violation feelings were positively related to the frequency of CWB-O/S; supporting Hypotheses 1a and 1b.

**Figure 3.**
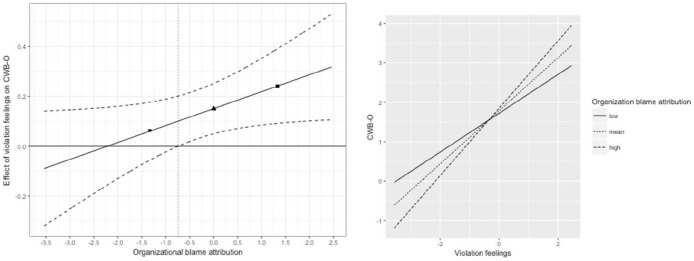
Johnson–Neyman plot (left side) and Aiken and West simple slope plot (right side) for the two-level moderating role of organizational blame attribution in the relationship between violation feelings and CWB-O. Note that the first symbol (circle) corresponds to low levels of organizational blame attribution (–1SD), the second symbol (triangle) corresponds to mean levels of organizational blame attribution (mean equals zero), and the third symbol (square) corresponds to high levels of organizational blame attribution (+1SD). In the Johnson–Neyman plot, organizational blame attributions significantly moderate the relationship between violation feelings and the frequency of CWB-O for any value on the right-hand side of the vertical dotted line.

Figure 3 shows the moderating role of organizational blame attributions on the relationship between violation feelings and CWB-O. The simple slopes of this moderating relationship were significant for any person-centered value of the organizational blame attributions above –.74. In simple slope terms, this means that as from medium levels of organizational blame attributions, a positive relationship exists between violation feelings and the frequency of CWB-O. This relationship became stronger as organizational blame attributions increased. Organizational blame attributions did not moderate the relationship between violation feelings and the frequency of CWB-S. These findings support Hypothesis 2.

**Figure 4.**
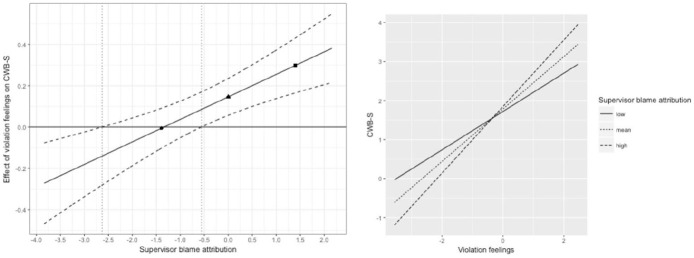
Johnson–Neyman plot (left side) and Aiken and West simple slope plot (right side) for the two-level moderating role of supervisor blame attribution in the relationship between violation feelings and CWB-S. Note that the first symbol (circle) corresponds to low levels of supervisor blame attribution (–1SD), the second symbol (triangle) corresponds to mean levels of supervisor blame attribution (mean equals zero), and the third symbol (square) corresponds to high levels of supervisor blame attribution (+1SD). In the Johnson–Neyman plot, supervisor blame attributions significantly moderate the relationship between violation feelings and the frequency of CWB-S for any value that is not captured between the vertical dotted line.

Figure 4 depicts the moderating role of supervisor blame attributions on the relationship between violation feelings and CWB-S. The simple slopes of this moderating relationship were significant for any person-centered value of organizational blame attributions below −2.63 and above −.56. In simple slope terms, this means that for extreme low values of supervisor blame attributions, violation feelings and CWB-S are negatively related to each other, whereas the relationship between violation feelings and CWB-S is positive as from medium levels of supervisor blame attributions. This relationship continues to become stronger as supervisor blame attributions increased. Supervisor blame attributions did not moderate the relationship between violation feelings and the frequency of CWB-O. These findings support Hypothesis 3.

Next, we found a significant time-lagged conditional indirect effect (estimate = .09; 95% CI = [.05; .13]) of the intensity of PCB on the frequency of CWB-O via violation feelings when blame was attributed to the organization. This effect was significant for medium to high values of organizational blame attributions. Similarly, we found a significant time-lagged conditional indirect effect (estimate = .08; 95% CI = [.05; .11]) of the intensity of PCB on the frequency of CWB-S via violation feelings for medium to high values of supervisor blame attributions. For both relationships this implies that the mediation effect grew stronger as blame attributions increased.

Finally, our results indicated that the frequency of CWB-O and CWB-S was positively related to perceived identity threat and organizational cynicism. However, only perceived identity threat was positively related to the intensity of PCB. We found a significant time-lagged indirect effect of the frequency of CWB-O (95% CI = [.01; .05]) and CWB-S (95% CI = [.01; .05]) on the intensity of PCB via perceived identity threat. We did not find significant time-lagged indirect effects of the frequency of CWB-O or CWB-S on the intensity of PCB via organizational cynicism.^3^ This implies that Hypotheses 4a and 4b were supported whereas Hypotheses 5a and 5b were not supported.

## General discussion

We aimed to identify a recursive relationship between PCB and CWB (Study 1) and explore two potential mechanisms underlying that relationship and the role of blame attributions as a crucial moderator of the PCB–CWB relationship (Study 2). Across both studies we found evidence for a recursive PCB–CWB relationship, indicating that PCB triggered acts of CWB, and that these acts of CWB triggered perceptions of PCB at a later point in time. These findings are in line with Cropanzano and Mitchell’s (2005) general conclusion that the output from one employee–employer transaction may serve as the input of the next employee–employer transaction, thus creating a cycle of actions and reactions. Moreover, we extended previous findings on the PCB–CWB relationship by highlighting the importance of blame attributions in an employee’s process of determining who is liable for action: the organization (CWB-O), or the supervisor (CWB-S). These findings are in line with a growing trend within the organizational justice, social exchange, and CWB literatures to acknowledge that employees direct different attitudes and behaviors toward multiple foci such as the organization and supervisors (for a review see Lavelle et al., 2007).

### Implications of research findings

This article extends research and theory on the harmful consequences of PCB in several important ways. First, both studies provide strong empirical evidence for the recursive PCB–CWB relationship among both volunteers and paid employees over different time periods (i.e., weekly and daily). These recursive relationships indicate that the relationship between PCB and CWB seems to linger and has the potential to become a vicious cycle with negative consequences for all parties involved. Although our findings concerning the PCB–CWB relationship are consistent with the general theoretical frameworks offered in existing PC literature, our findings concerning the CWB–PCB relationship broaden the theoretical explanations beyond those offered in the existing PC literature.

Drawing on Self-Consistency Theory (Korman, 1970), we demonstrate that current acts of CWB-O/S may not be perceived as consistent with one’s image of a moral person. Therefore, it seems that one will seek information in support of the idea that these acts of CWB-O/S were justified by focusing on the fact that the organization breached the PC in the past and most likely will continue to do so in the future. At the same time, employees who have engaged in CWB-O/S may have developed a negative self-view following the enactment of CWB and as a consequence seek out negative feedback. By arguing for, and empirically demonstrating, this reciprocal PCB–CWB relationship, we showed that changes in cognitions play an important role in unfolding PCB reactions, next to more affective (i.e., feelings of violation) and social exchange (i.e., changes in delivered inducements and contributions) explanations. Moreover, although theoretical attention has been given to the PC as a dynamic process (Hansen and Griep, 2016; Tomprou et al., 2015), little conceptual and theoretical attention has been given to the recognition of behavior *as both an outcome and antecedent* of PCB, as well as the incorporation of feedback loops from these behaviors to work experiences and the other way round. By recognizing this role of time and processes – rather than (semi) static snapshots – we are better equipped to explain how individuals interpret and react to these dynamic and evolving events. Specifically, we propose to abandon a static way of thinking about the PCB–CWB relationship (one in which CWB is always the outcome of PCB) to make room for a more dynamic way of thinking in which we account for the option that current acts of CWB may not only happen in reaction to past perceptions of PCB but may also serve as antecedents to future perceptions of PCB.

Our second major extension to PC research and theory concerns a reconceptualization of employees’ retaliation process. While a substantial body of research has focused on the presumed positive relationship between perceptions of PCB or experienced violation feelings, and acts of counterproductivity, these studies did not account for the proposition that employees will direct their corrective action to the source of frustration (i.e., the organizational agent breaking its obligations; see Lavelle et al., 2007). Moreover, beyond a handful of studies focusing on attributions (e.g., Chao et al., 2011; Conway et al., 2014; Turnley et al., 2003), the role of attributions has largely been overlooked. However, our results indeed indicated that an employee is more likely to direct a behavioral reaction toward the source that is blamed for the resulting negative feelings. Our findings provide support for the assumption that an employee will retaliate against the entity with whom a PC exists, and they underline the need to explicitly assess the extent to which casting blame might explain differences in an employee’s retaliation process (see also Lavelle et al., 2007).

### Limitations

Like all studies, our research has limitations that deserve further attention. First, we collected all variables at the same point in time by means of repeated measurement surveys. This might raise concerns with common method variance at each time point (Podsakoff et al., 2012). However, by using weekly and daily time lags, we reduced risks owing to common method bias. In addition, we presented all scales in a random order, both within and between blocks. Finally, Siemsen and colleagues (2010) argued that common method bias cannot explain or distort interaction effects. Hence, the presence of these significant interactions in our studies helps to strengthen our argument that the observed relations are a function of the studied constructs and relationships rather than methodological artifacts.

Although we used well-accepted PCB measures, we are unable to assess the extent to which different PC inducements were promised versus the extent to which these inducements were actually delivered. This could be an important limitation to keep in mind because previous studies demonstrated that satisfaction was higher (Lambert et al., 2003) and violation feelings were lower (Montes and Irving, 2008) among employees who were promised and received high levels of inducements compared to those who were promised and received low levels of inducements. In this respect, we would like to direct attention to the work of Lambert and colleagues (2003) as they demonstrated that measuring promised and delivered inducements separately, and conducting polynomial regression with response surface analysis partially overcomes this limitation.

### Suggestions for future research

First, there may be important between- and within-person fluctuations in the extent to which one perceives a discrepancy as a PCB. According to Schalk and Roe (2007) a discrepancy will only be noticed (and perceived as a PCB) when it exceeds a certain threshold or personal zone of acceptance. This mechanism has implications for the recursive PCB–CWB relationship because when one has a lower threshold sensitivity, one will be more likely to interpret a deviation as a PCB, which in turn sets the negative recursive relationships between PCB and CWB into motion more easily compared to incidences were one has a higher threshold sensitivity. The extent to which one perceives a discrepancy as a PCB could be investigated by means of a repeated measurement design in which the data are analyzed using spline regression. In this type of analysis, the slope of the regression line changes for different ranges of the independent variable. The values where the slope of the regression line changes is represented by knots which reflect the threshold at which one will move from ‘not perceiving a discrepancy as a PCB’ to ‘perceiving a discrepancy as a PCB’ (Seber and Wild, 2003).

Second, while our current two-study article identifies the recursive PCB–CWB relationship, we did not look into specific ways to overcome this negative relationship. Future research could benefit from studying mechanisms that might redress the behavioral consequences of PCB and violation feelings as well as mitigate the attitudinal consequences of engaging in CWB-O/S. Based on the limited theoretical (see Tomprou et al., 2015) and empirical (see Tomprou et al., 2016) work on the aftermath of PCB, we would advise future research to look into organizational actions (e.g., recognizing a PCB, providing a clarification and taking corrective action), the quality of the employee–employer relationship (social support and caring for one’s well-being), peer and supervisor involvement (speak up on behalf of the employee), and proactive employees’ actions (take actions to renegotiate an alternative PC or seek advice or other instrumental aid from others) as mechanisms that could potentially break down the recursive PCB–CWB relationship. In an exploratory interview study by Tomprou and colleagues (2016) these factors were found to influence the extent to which employees believed that a PCB and its ensuing violation feelings were resolved, as well as the extent to which they were satisfied with the resolution process.

### Practical implications

Two main practical interventions become evident while unraveling the complex role of CWB-O and CWB-S as both a consequence and antecedent of PCB. Given the demonstrated PCB–violation feelings–CWB relationship, we suggest that organizational agents provide social and emotional support (e.g., care for one’s well-being, listen to one’s complaints and speak up on behalf of employees) to employees who perceive a PCB because this has been linked to successful re-establishment of a meaningful and positive reciprocal exchange relationship (Maitlis and Lawrence, 2007). In line with the theoretical assumptions of Tomprou and colleagues (2015), perceiving support signals an organization’s willingness to resolve the PCB. As a consequence, an employee who perceives that organizational agents are (increasingly) supportive might develop more positive perceptions following work-related negative experiences such as perceptions of PCB compared to their less supported counterparts (e.g., Van Dyne et al., 2003). On the employee side, we argue that the use of reappraisal, in contrast to suppression, strategies might mitigate the positive PCB–CWB relationship (Gross and John, 2003). Specifically, by using reappraisal strategies the employee might re-construe the situation in a way that it changes the emotional impact. For example, the employee might reinterpret the situation as one wherein no willful PCB happened (e.g., potentially there were reasons beyond the organization’s control that might explain the PCB).

Next, given the escalating nature of the recursive PCB–CWB relationship, we would like to emphasize that *timeliness* is a crucial factor in resolving CWB. In this case, timeliness refers to the discordance between an employee’s perceived speed of discrepancy reduction (i.e., the perceived speed at which an organization delivers intervention or remedy) and the desired speeds of discrepancy reduction (i.e., the speed at which an employee desires intervention or remedy). Building on recent work by Tomprou and colleagues (2015), we argue that the longer an organization or supervisor delays any form of intervention, the more likely it becomes for perceptions of PCB, violation feelings and acts of CWB to persist or even intensify over time. As a consequence, a slow resolution velocity might result in downward spirals of negative attitudinal or behavioral reactions over time. In contrast, a positive discordance between an employee’s preferred speed of discrepancy reduction and the perceived speed of discrepancy reduction (i.e., the organization or supervisor intervenes faster or equally fast to an employee’s desired speed of discrepancy reduction) increases the likelihood that an employee believes that the organization or supervisor is aware of its wrongdoing, sufficiently concerned about the mutual exchange relationship, and willing to make amends. Hence, it seems advisable for organizational agents to intervene as soon as they notice that an organizational obligation is breached. However, for this to happen, organizational agents should be aware of the extent to which an employee perceives a specific inducement as an obligation on behalf of the organization. Therefore, we advise organizational agents to communicate clearly about what they can and cannot offer to an employee in return for certain contributions on their behalf. By communicating clearly about what both parties bring to the table, they can develop and update their mutual obligations and contributions.

## Conclusion

Across both studies we found evidence for a PCB–CWB relationship, indicating that perceptions of PCB may trigger enactment of CWB at a later point in time via violation feelings, and that these acts of CWB may trigger perceptions of PCB at a later point in time among both voluntary workers and paid employees. Moreover, we identified in Study 2 that this recursive CWB–PCB relationship is mediated by perceived identity threat. Finally, we extended previous findings on the PCB–CWB relationship by highlighting the importance of blame attributions in an employee’s process of determining who is liable for action: the organization (CWB-O) or the supervisor (CWB-S).

## Appendix 1 – Study 1

*Perceptions of psychological contract breach* were measured with 20 items by PSYCONES (2005), Kickul and Lester (2001), Robinson and Rousseau (1994), Robinson and Morrison (1995), Rousseau (1990) and Bingham (2005). In line with the recommendations by Conway and Briner (2002) and Griep and colleagues (2016), we asked respondents to indicate (‘yes’ or ‘no’) whether their organization had breached its obligations toward them for each of these PC inducements (i.e., ‘Did your organization breach one or more of the following obligations during the past week?’). The items were:

1. Providing a good working atmosphere
2. Ensuring fair treatment by managers and supervisors
3. Helping in dealing with problems encountered outside work
4. Providing a safe working environment
5. Providing a work environment free from violence and harassment
6. Providing interesting work
7. Providing opportunities to advance and grow
8. Allowing to take part in decision making
9. Providing challenging work
10. Improving future prospects
11. Providing career opportunities within the work
12. Contribute to the stated cause
13. Commit resources toward advancing the stated cause
14. Stand behind our corporate ideology, even if it requires a financial sacrifice
15. Be dedicated to my company’s mission
16. Provide opportunities for involvement in our cause
17. Encourage employee involvement in the cause
18. Create internal practices and policies that advance my company’s ideals
19. Act as a public advocate of the espoused cause
20. Maintain company culture that promotes our corporate principles

*Feelings of violation* were measured with the single item by Solinger and colleagues (2016):

1. To what extent did the breach of this (these) obligation(s) have a negative emotional effect on you during the past week?

*CWB-O* was measured with the following six items by Dalal and colleagues (2009):

1. Did not work to the best of my ability
2. Spent time of tasks unrelated to work
3. Criticized organizational policies
4. Took an unnecessary break
5. Worked slower than necessary
6. Spoke poorly about my organization to others

### Appendix 1 – Study 2

*Perceptions of psychological contract breach* were measured with the following five items by Robinson and Morrison (2000):

1. Almost all the promises made by my employer during recruitment have been kept so far (reverse)
2. I feel that my employer has come through in fulfilling the promises made to me when I was hired (reverse)
3. So far my employer has done an excellent job of fulfilling its promises to me (reverse)
4. I have not received everything promised to me in exchange for my contributions
5. My employer has broken many of its promises to me even though I’ve upheld my side of the deal

*Feelings of violation* were measured with the following four items by Robinson and Morrison (2000):

1. I feel a great deal of anger toward my organization
2. I feel betrayed by my organization
3. I feel that my organization has violated the contract between us
4. I feel extremely frustrated by how I have been treated by my organization

*Organizational blame attributions* were measured by presenting the same 5-item PCB scale by Robinson and Morrison (2000) and asking our respondents to indicate on a 7-point scale, ranging from (1) ‘minimally or not at all’ to (7) ‘to a very great extent’, the extent to which they blamed their (1) organization and (2) supervisor:

1. I blame my organization for the fact that almost none of the promises made by my employer during recruitment have been kept so far
2. I blame my organization for the fact that I feel that my employer has not come through in fulfilling the promises made to me when I was hired
3. I blame my organization for the fact that so far my employer has not done an excellent job of fulfilling its promises to me
4. I blame my organization for the fact that I have not received everything promised to me in exchange for my contributions
5. I blame my organization for the fact that my employer has broken many of its promises to me even though I’ve upheld my side of the deal

*Supervisor blame attributions* were measured by presenting the same 5-item PCB scale by Robinson and Morrison (2000) and asking our respondents to indicate on a 7-point scale, ranging from (1) ‘minimally or not at all’ to (7) ‘to a very great extent’, the extent they blamed their (1) organization and (2) supervisor:

1. I blame my supervisor for the fact that almost none of the promises made by my employer during recruitment have been kept so far
2. I blame my supervisor for the fact that I feel that my employer has not come through in fulfilling the promises made to me when I was hired
3. I blame my supervisor for the fact that so far my employer has not done an excellent job of fulfilling its promises to me
4. I blame my supervisor for the fact that I have not received everything promised to me in exchange for my contributions
5. I blame my supervisor for the fact that my employer has broken many of its promises to me even though I’ve upheld my side of the deal

*CWB-O* was measured with the following six items by Dalal and colleagues (2009):

1. Did not work to the best of my ability
2. Spent time of tasks unrelated to work
3. Criticized organizational policies
4. Took an unnecessary break
5. Worked slower than necessary
6. Spoke poorly about my organization to others

*CWB-S* was measured with the following six items by Dalal and colleagues (2009):

1. Behaved in an unpleasant manner toward my supervisor
2. Tried to harm my supervisor
3. Criticized my supervisor’s opinion or suggestion
4. Tried to avoid interacting with my supervisor
5. Excluded my supervisor from a conversation
6. Spoke poorly about my supervisor to others

*Identity threat* was measured with the following four items by Breakwell (1986):

1. I felt that my sense of self-worth was undermined
2. I felt that it makes me feel less competent
3. I felt that my identity has changed
4. I felt that it makes me feel less unique as a person

*Organizational cynicism* was measured with the following 12 items by Dean and colleagues (1998):

1. I believe that my company says one thing and does another
2. When I think about my company, I feel a sense of anxiety
3. My company expects one thing of its employees, but rewards another
4. When I think about my company, I experience aggravation
5. We look at each other in a meaningful way with my colleagues when my institution and its employees are mentioned
6. When I think about my company, I experience tension
7. When I think about my company, I get angry
8. I criticize the practices and policies of my company to people outside the hospital
9. In my company I see very little resemblance between the events that are going to be done and the events which are done
10. My company’s policies, goals and practices seem to have little in common
11. I talk with others about how work is being carried out in the company
12. If an application was said to be done in my company, I’d be more skeptical whether it would happen or not

## Notes

1. Colleagues are not typically considered responsible for upholding the PC between an employee and his/her organization, making co-workers unlikely targets of retaliation by employees who perceived a PCB. Therefore, we focus only on CWB-O and CWB-S throughout the remainder of this article.

2. Only when they engaged in voluntary activities for at least one day during the past week was that person asked to reflect upon the past week when filling out the weekly survey. In all other cases, respondents were not required to complete the survey and the data were treated as missing.

3. It is noteworthy that – when estimated in separate models – both perceived identity threat and organizational cynicism mediated the relationship between CWB-O and PCB (time-lagged indirect effect for organizational cynicism and perceived identity threat are 95% CI = [.02; .05] and 95% CI = [.01; .03], respectively) and the relationship between CWB-S and PCB (time-lagged indirect effect for organizational cynicism and perceived identity threat are 95% CI = [.01; .05], and 95% CI = [.02; .06], respectively).
